# The Role of PI3K/Akt/mTOR Signaling in Gastric Carcinoma

**DOI:** 10.3390/cancers6031441

**Published:** 2014-07-07

**Authors:** Tasuku Matsuoka, Masakazu Yashiro

**Affiliations:** 1 Department of Surgical Oncology, Osaka City University Graduate School of Medicine, 1-4-3 Asahi-machi, Abeno-ku, Osaka 545-8585, Japan; E-Mail: m4808279@med.osaka-cu.ac.jp; 2 Oncology Institute of Geriatrics and Medical Science, Osaka City University Graduate School of Medicine, 1-4-3 Asahi-machi, Abeno-ku, Osaka 545-8585, Japan

**Keywords:** PI3K/Akt/mTOR pathway, gastric carcinoma, target therapy, apoptosis, matastasis, chemo-resistance

## Abstract

The phosphatidylinositol 3-kinase (PI3K)/Akt/mammalian target of rapamycin (mTOR) pathway is one of the key signaling pathways induced by various receptor-tyrosine kinases. Accumulating evidence shows that this pathway is an important promoter of cell growth, metabolism, survival, metastasis, and resistance to chemotherapy. Genetic alterations in the PI3K/Akt/mTOR pathway in gastric carcinoma have often been demonstrated. Many kinds of molecular targeting therapies are currently undergoing clinical testing in patients with solid tumors. However, with the exception of the ErbB2-targeting antibody, targeting agents, including PI3K/Akt/mTOR inhibitors, have not been approved for treatment of patients with gastric carcinoma. This review summarizes the current knowledge on PI3K/Akt/mTOR signaling in the pathogenesis of gastric carcinoma and the possible therapeutic targets for gastric carcinoma. Improved knowledge of the PI3K/Akt/mTOR pathway in gastric carcinoma will be useful in understanding the mechanisms of tumor development and for identifying ideal targets of anticancer therapy for gastric carcinoma.

## 1. Introduction

Gastric carcinoma remains the second most common cause of cancer-related deaths in the world. Although the outcomes of gastric carcinoma have recently improved, highly malignant type of gastric carcinoma, which is characterized by rapid progression and resistance to chemotherapy, continue to have poor prognosis [1].

To date, progress in the understanding of molecular pathways involved in a variety of cancers has led to the discovery of new targeted therapies, including therapies for gastric carcinoma. Trastuzumab has already been approved as a standard therapy for human epidermal growth factor receptor 2 (HER2)-positive gastric carcinoma patients, on the basis of results of the Trastuzumab for Gastric Cancer clinical trial [2]. Further candidate molecules related to cell growth, invasion, apoptosis, angiogenesis, and metastasis are emerging as a new strategy for the treatment of gastric carcinoma. Agents targeting molecules such as epidermal growth factor receptor (EGFR), vascular endothelial growth factor (VEGF) receptor, and fibroblast growth factor receptor (FGFR), as well as pathways such as c-Met pathways, are promising candidates for targeted therapy for gastric carcinoma and are now in clinical development [3,4].

It is known that accumulation of genetic alterations results in the development of gastric carcinoma [5]. Most of the genetic mutations in gastric carcinoma correlate with changes in biological signals, such as those in the phosphatidylinositol 3-kinase/Akt/mammalian target of rapamycin pathway (PI3K/Akt/mTOR pathway) [6]. PI3K signaling is a crucial regulator of many essential cellular processes, including cell growth, metabolism, survival, metastasis, and resistance to chemotherapy [7]. Although there is substantial evidence that the PI3K/Akt/mTOR pathway is frequently altered in gastric carcinoma, its precise function remains to be determined [7,8,9]. Understanding the biological pathways leading to the development of gastric carcinoma will provide an opportunity to enhance targeted therapies. In this context, we review the role of PI3K signaling in gastric carcinoma, especially on carcinogenesis, growth, adhesion, metastasis, apoptosis, and sensitivity to therapy. Novel therapies targeting the PI3K/AkT/mTOR pathway as well as its downstream substrates are summarized with the literatures in these several decade.

## 2. The PI3K/Akt/mTOR Pathway

**Figure 1:**
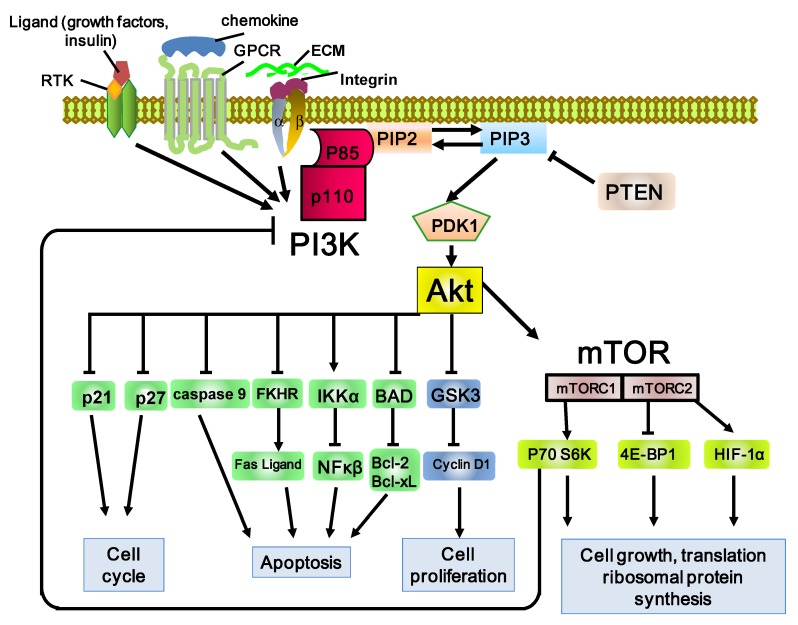
Schematic representation of the PI3K/Akt/mTOR pathway. The PI3K pathway involves many factors, including the binding of receptor tyrosine kinase (RTK), G-protein-coupled receptors (GPCR), and GTP-binding proteins to adaptor proteins. PI3K consists of the catalytic subunit, p110, and the regulatory subunit, p85. PI3K phosphorylates PIP2 (phosphatidylinositol 3,4-bisphosphate) and produces PIP3 (phosphatidylinositol 3,4,5-trisphosphate). PIP3 then activates 3-phosphoinositide-dependent kinase 1 (PDK1) and its major downstream effector, Akt. Phosphorylation of Akt promotes cell proliferation, survival, migration, and differentiation. Phosphatase and tensin homolog (PTEN) dephosphorylates PIP3 and inhibits activation of Akt by PIP3. Phosphorylation of Akt induces the activation of one of the major downstream effectors, mTOR (mammalian target of rapamycin). mTOR phosphorylates S6K1 and 4EBP1, directly leading to increased translation and synthesis of cell-cycle-regulating and ribosomal proteins. Stimulatory events are indicated by arrows and inhibitory events are indicated by lines ending in flat lines.

The PI3K pathway (Figure 1) has many different upstream factors, including the binding of receptor tyrosine kinase (RTK), G-protein-coupled receptors (GPCR), and GTP-binding proteins to adaptor proteins [7,10]. PI3K belongs to a lipid kinase family characterized by their ability to phosphorylate the 3'-OH group of the inositol ring in inositol phospholipids [10]. They are categorized into three classes, namely, classes I, II, and III. Class I PI3Ks are further divided into class IA and IB. The catalytic subunit of class IA PI3K, p110, has four isoforms (p110α, p110β, p110γ, and p110δ), whereas its p85 regulatory subunit has three variants (p85α, p85β, and p55γ). The isoforms of p110 are encoded by the *PIK3CA*, *PIK3CB*, and *PIK3CD* genes. Deregulation of the PI3K/Akt/mTOR pathway can occur subsequent to oncogenic mutations of *PIK3CA* [11]. p110α is the most well-understood isoform in terms of signal transduction and physiological association and the most often mutated or amplified in solid tumors [12]. PI3K activation usually occurs through growth factor stimulation by phosphotyrosine kinases such as EGFR, platelet-derived factor receptor, insulin growth factor receptor, or c-Met. Activated PI3K associates with the receptor through one or two Src homology 2 domains in the regulatory subunit, which leads to the activation of the catalytic subunit. Activation of the PI3K pathway leads to the phosphorylation of the inositol ring of lipids in the plasma membrane and converts phosphatidylinositol 3-phosphate (PI) and phosphatidylinositol 4,5-bisphosphate (PIP2), the lipid substrates for class I PI3Ks, to phosphatidylinositol 3,4,5-trisphosphate (PIP3). PIP2 and PIP3 interact with pleckstrin homology (PH) domain-containing proteins on the inner surface of the plasma membrane, resulting in conformational changes of these proteins.

PH domains are found in many proteins, including Akt, which is also known as protein kinase B [13]. Akt is a serine–threonine kinase that normally exists in the cytoplasm. Recently, three members of the Akt family, namely, Akt1, Akt2, and Akt3, have been isolated. These are products of three distinct genes that share up to 80% homology at the amino acid level. Upon activation of PI3K, Akt transfers to the cell membrane, resulting in its conformational change. Akt contains a central kinase domain with a threonine residue (T308) that binds to the phosphoinositide-dependent protein kinase 1 (PDK1) and a C-terminal tail domain (S473) that binds to the second mTOR complex 2 (mTOR2). Phosphorylated Akt (p-Akt) has been shown to promote molecular functions within the cell, such as cell cycle progression and angiogenesis, as well as prevent apoptosis through a number of downstream effectors [14]. Glycogen synthase kinase 3 (GSK3), the first identified Akt substrate, is believed to be an essential metabolic enzyme and an important factor in other signaling cascades. It phosphorylates a host of downstream substrates such as p21, p27, caspase 9, FKHR, IKKα, and BAD, thereby mediating a number of effects [15]. PI3K activity is regulated by the lipid phosphatase and tensin homolog (PTEN), a tumor suppressor gene that encodes a lipid phosphatase that downregulates the PI3K signal by converting PIP3 back to PIP2 [16]. Loss of PTEN results in constitutive activation of Akt and in alteration of downstream factors in Akt signaling. 

mTOR is a highly conserved protein kinase that participates as an effector in the PI3K/Akt pathway. mTOR comprises two protein complexes, mTORC1 (mTOR, mLST8, and raptor) and mTORC2 (mTOR, mLST8, mSIN1, and Rictor). mTOR1, a complex that is also modulated by extracellular-signal-regulated kinase, induces protein synthesis and cell growth by regulating ribosomal p70S6 kinase 1 (S6K1) and eukaryotic translation factor 4E-binding protein 1 (4EBP1) [17]. Activated S6K1 participates in negative feedback, thereby attenuating activation of the PI3K pathway through phosphorylation and subsequently inhibiting adaptor molecule insulin receptor substrate 1, which interrupts the signaling between IGF-1 and PI3K. mTORC2 phosphorylates Akt and SGK1 at the C-terminal and regulates the remodeling of the actin cytoskeleton, but the biological significance of these activities is still largely unknown [18]. mTOR plays a critical role in the regulation of tumor cell motility and cancer metastasis [19]. However, the underlying mechanism of mTOR regulation of cell motility and mTOR inhibitors inhibiting tumor cell motility is controversial [20].

## 3. Gene Mutation and Activation of PI3K/Akt/mTOR Pathway in Gastric Carcinoma

Genetic alterations in the PI3K/Akt/mTOR pathway in gastric carcinoma have been demonstrated frequently. The alterations and activity founded in gastric carcinoma are summarized in Table 1. These events are due to several mechanisms, including mutation and amplification of genes encoding crucial components of the signaling cascade.

The catalytic subunit *PIK3CA* gene is mutated at a high frequency in gastric carcinoma cell lines and tumor tissues [11]. Several studies have demonstrated that somatic mutations in *PI3KCA* are present in 4%–25% of patients with gastric carcinoma [21,22,23,24,25]. The mutation incidence is high (21.4%) in T4 cancers and low (6.4%) in T2 cancers. Therefore, *PIK3CA* mutations are likely to be late events in gastric carcinogenesis [24]. Although *PIK3CA* mutations preferentially occur together with activating KRAS-BRAF mutations in colorectal carcinoma, *PIK3CA* mutations tend to occur as isolated events, such as those in mismatch repair deficiency in gastric carcinoma [21]. In contrast, another study showed that *PIK3CA* mutations are rare, but their amplification is very common in gastric carcinoma. Therefore, amplification could be a major mechanism in activating the PI3K/Akt pathway in this type of malignancy [26]. The *PIK3R3* gene, which encodes the PI3K regulatory subunit p55γ, was found to be significantly upregulated in gastric tumor specimens. Silencing *PIK3R3* resulted in reduction of the growth of gastric carcinoma cells, induction of G0/G1 cell-cycle arrest, as well as attenuation of retinoblastoma protein phosphorylation and of cyclin D1 and PCNA expression [9].

High expression levels of Akt and p-Akt were observed in 74% and 78% of gastric tumors, respectively [27]. p-Akt expression was significantly correlated with the depth of invasion, number of lymph nodes, and poor prognosis in gastric carcinoma with respect to tumor angiogenesis [28,29]. On the other hand, p-Akt expression was also significantly associated with HER2 overexpression but not with *PIK3CA* mutations [30]. A survey of 225 human tumors for changes involving *AKT1* led to the discovery of a 20-fold amplification of this gene in gastrointestinal tumors [31]. Additionally, high activity of GSK3β was found to be frequently present in early-stage gastric carcinoma and was positively associated with good prognosis. Thus, GSK3β could be a useful prognostic marker for gastric carcinoma [32]. 

The tumor suppressor gene *PTEN* located on chromosome 10q23.3 acts as a plasma membrane lipid phosphatase. Several studies revealed that inactivation of PTEN is closely correlated with the initiation and development of gastric carcinoma. There are reports that PTEN could be a prognostic biomarker for this type of malignancy. A direct-sequencing study indicated 27 cases with mutations among 144 patients (18.8%) consisting of 15 cases of missense mutations, nine nonsense mutations, two 1-bp deletions, and a mutation in intron 6. The mutation status of *PTEN* was significantly related to pTNM staging and to the degree of cell differentiation [33]. The importance of the loss of function of PTEN in metastatic gastric carcinomas is well described and its role includes homozygous deletions, loss of heterozygosity, and inactivation of mutations [34]. Interestingly, the lack of PTEN in gastric carcinoma is more often found in the cardia than in other areas of the stomach [34]. In addition to amplification of the Akt1 gene locus, frequent monoallelic deletions of PTEN phosphatase antagonism of PI3K/Akt in 47% of cases [35] or of promoter methylation in 39% of cases have been described [36]. An immunohistochemical study showed positive correlations between mTOR expression in gastric carcinomas and pathological parameters such as invasive depth, differentiation, and lymph node metastasis. The positive rate of mTOR expression was found to be much higher in tumor cells, whereas little or no expression in normal gastric tissues was observed [37]. This report also showed that the expression levels of mTOR and PTEN were negatively correlated in the PI3K/Akt/mTOR signaling pathway.

Several other well-known upstream oncogenes such as those that activate mutations in RAS family members, as well as mutations and amplifications of RTKs (e.g., EGFR and HER2/neu) mediate their effect in part through activation of PI3K signaling. Interestingly, the prevalence of these events are depending on the carcinoma type. As there is evidence that various mutations are non-equivalent in their functional dependencies, clarifying the specific oncogenic events that are present in specific cancers is critical [38]. 

**Table 1:** Genetic alterations and activities of the PI3K/Akt/mTOR pathway in gastric carcinoma.

Study [Ref#]	PMID	Sample	Main Results
*PI3K*			
Samuels *et al.* [11]	15016963	Tumor specimens	Mutations in *PIK3CA* were identified in 3 of 12 gastric cancers (25%).
Velho *et al.* [21]	15994075	Tumor specimens	*PIK3CA* mutations in exons 9 and 20 were present in 10.6% of gastric carcinomas.
Barbi *et al.* [23]	20398348	Tumor specimens	*PIK3CA* mutations were present in 16% of gastric carcinomas. No other association between *PI3KCA* mutations and their clinical pathological covariates was found.
Sukawa *et al.* [24]	24458107	Tumor specimens	The mutation incidence is high (21.4%) in T4 cancers and low (6.4%) in T2 cancers.
Corso *et al.* [25]	20937558	Tumor specimens	Mutations in *PIK3CA* gene occurred in 14.3% of the MSI gastric cancers.
Shi *et al.* [26]	22292935	Tumor specimens	*PIK3CA* mutations are rare, but their amplification is very common in gastric carcinoma.
Zhou *et al.* [9]	22876838	Cell lines/tumor specimens	PIK3R3 was significantly up-regulated in gastric cancer specimens, and 9.5% to 15% tumors showed more than 2 fold increase compare to the paired mucosa tissues.
***Akt***			
Num *et al.* [27]	14678019	Tumor specimens	Akt expression was detected in 74% of the tumors and pAkt expression in 78%.
Cinti *et al.* [28]	18841391	Tumor specimens	There was a statistically significant correlation between pAkt expression and depth of infiltration of the tumor, number of infiltrated lymph nodes and p34/cdc2 expression.
Kobayashi *et al.* [29]	16785763	Cell lines/tumor specimens	pAkt expression was detected in 57% of the tumors, which was correlated with high clinicopathological parameters as well as a poor outcome.
Sukawa *et al.* [30]	23236232	Tumor specimens	pAkt expression was also significantly associated with HER2 overexpression but not with *PIK3CA* mutations
Staal *et al.* [31]	3037531	Cell lines/tumor specimens	A survey of 225 human tumors for changes involving AKT1 led to the discovery of a 20-fold amplification of this gene in one of the five gastric adenocarcinomas tested.
Cho *et al.* [32]	20704706	Cell lines/tumor specimens	High activity of GSK3β was found to be frequently present in early-stage gastric carcinoma and was positively associated with good prognosis.
***PTEN***			
Wen *et al.* [33]	20514448	Tumor specimens	*PTEN* mutations were present in 55.6% of missense mutation, 33.3% of nonsense mutation, 7.4% of 1-bp deletion and 3.7% of a mutation within intron.
Mina *et al.* [34]	22639407	Tumor specimens	4.4% of primary gastric cancer spots showed PTEN deletions. PTEN deletion was correlated with nodal and distant metastases.
Byun *et al.* [35]	12569555	Cell lines/tumor specimens	Frequent monoallelic deletions of PTEN phosphatase antagonism of PI3K/Akt in 47% of cases.
Kang *et al.* [36]	11896207	Tumor specimens	The promoter methylation frequency of PTEN was found to be present in 39% of cases examined, and 73% of gastric cancer tissues showing promoter methylation exhibited the loss of PTEN expression.
***mTOR***			
Li *et al.* [37]	23205120	Tumor specimens	The expression levels of mTOR and PTEN were negatively correlated in the PI3K-AKT-mTOR signaling pathway.

## 4. The Role of the PI3K/Akt/mTOR Pathway in the Biological Properties of Gastric Carcinoma

### 4.1. Apoptosis

**Figure 2:**
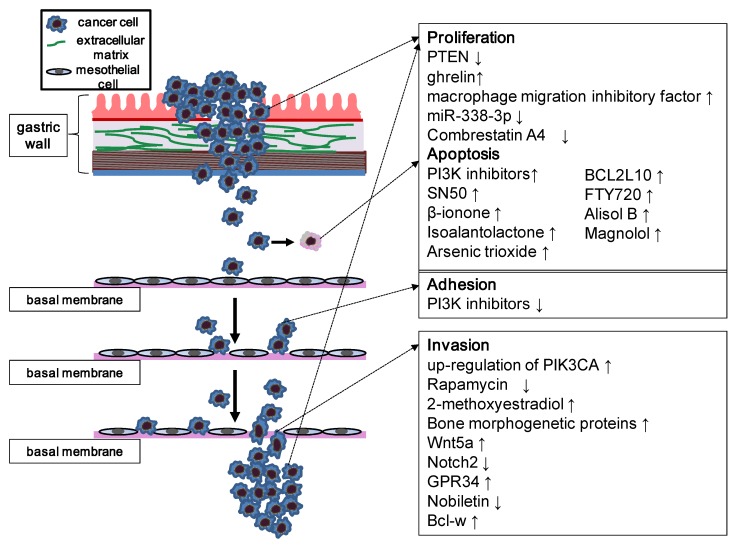
Schematic representation of the PI3K/Akt/mTOR pathway involved in carcinoma metastasis. The figure focuses on the biological process of peritoneal metastasis, which is one of the most common metastatic types of gastric carcinoma, although these principles apply to most patterns of metastases. The PI3K/Akt/mTOR pathway is involved in each step of metastasis; thus, numerous inhibitors and agents can affect these processes by modulation of PI3K signaling. Stimulatory events are indicated by upward arrows and inhibitory events are indicated by downward arrows.

The anti-apoptotic activities of the PI3K/Akt/mTOR pathway are closely related to the resistance of the malignant cells to a spectrum of apoptosis-stimulating factors (Figure 2). Several studies showed that the use of PI3K inhibitors substantially facilitates apoptosis [39]. A recent investigation demonstrated that PI3K inhibition induces mitochondrial function and upregulates p53 and PUMA levels [40]. A synthetic small-molecule pan-PI3K inhibitor, LY294002, significantly inhibits the growth of gastric carcinoma while promoting apoptosis, resulting in the downregulation of MMP-2, MMP-9, and VEGF [41]. Impairment of PI3K/Akt activity attenuates the activity of κ-light-chain enhancer of activated B cells (NF-κB) and increases apoptosis in gastric carcinoma cells. This effect induced by blocking PI3K/Akt was ascribed to the inhibition of NF-κB activity through IKK/IκB [42]. Furthermore, the NF-κB inhibitor SN50 leads to an increase in the effect of LY294002 on inducing death of human gastric carcinoma cells through upregulated expression of p53, PUMA, and Beclin1 [43]. Several factors have been reported to induce apoptosis via the PI3K/Akt pathway in gastric carcinoma [44,45,46,47,48]. Apoptosis of gastric carcinoma cells induced by isoalantolactone was associated with the dissipation of mitochondrial membrane potential due to downregulation of Bcl-2 and upregulation of Bax [49]. Some studies have shown that PTEN could enhance Fas/FasL or cytochrome c-mediated apoptosis that involves activation of caspase-3, suggesting that decreased PTEN expression could downregulate caspase-3, thus disrupting the apoptotic pathway in gastric carcinoma [50].

### 4.2. Metastasis

More than half of patients with advanced gastric carcinoma have distant metastasis upon its diagnosis [1]. To date, no molecular indicator is available for predicting metastasis in this disease. However, recent studies have shown that p-Akt is highly expressed in the lymph node and in distant metastases compared with that in the primary gastric tumor, suggesting that Akt activity plays a role in promoting metastasis in gastric carcinoma patients [51]. p-Akt positivity also correlates with microvessel density and VEGF, suggesting that p-Akt and VEGF have a significant role in angiogenesis in gastric adenocarcinoma [52]. Dominant-negative Akt inhibits the proliferation of gastric carcinoma cells and induces G1 cell cycle arrest, whereas upregulation of Akt increases cell proliferation [53]. On the other hand, gastric carcinoma tissues strongly express PI3K p85α and p-AkT, and targeted blockade of the PI3K pathway inhibits gastric carcinoma growth and metastasis through downregulation of Ki-67 and MMP-2 expression [54]. Combrestatin A4, an anti-angiogenic compound, reduces the growth of a cell line with high Akt expression. Downregulated Akt suppresses tumor formation and metastasis by reducing cell attachment, migration, and invasion [55]. The PI3K/Akt pathway also enhances the adhesiveness of metastatic gastric carcinoma cells. Our previous data reveal that attachment of OCUM-2MD3 cells, an extensively peritoneal-seeding human scirrhous gastric cancer cell line, to type IV collagen increased Akt activation; whereas PI3K/Akt inhibition decreased adhesion of in a dose-dependent manner. Immunoprecipitation studies indicated that the PI3K/Akt pathway is associated with integrin signaling through Src and vinculin, leading to cytoskeletal reorganization [56]. 

Upregulation of *PIK3CA* expression is likely related to lymph node metastasis in gastric cancer [51]. Upregulated *PIK3CA* enhances the activity of PI3K/Akt signaling through increased activity of PI3K p110α, thereby promoting the invasion and metastasis of gastric carcinoma cells [51]. Downregulation of *PIK3CA* in these cells reduces their ability to proliferate, migrate, and invade. Robust knockdown of *PIK3CA* by siRNA results in decreased catalytic activity of PI3K and subsequent dephosphorylation of Akt in these cells [57]. PI3K signaling regulates Rac1 activity, resulting in the induction of hypoxia-inducible factor 1α and VEGF [58]. 

PTEN-mediated dephosphorylation of FAK can influence the growth and invasion of gastric carcinoma [59]. Rapamycin has been reported to inhibit the migration of gastric cancer cells. Chemokine (CXC motif) ligand 12 (CXCL12) could be an independent prognostic factor in gastric carcinoma. The mTOR pathway was also found to play an important role in CXCL12/CXCR4-mediated cell migration [60].

Several factors mediate the metastatic capacity of gastric carcinoma through the PI3K/Akt/mTOR pathway [61,62,63,64,65]. Administration of 2-methoxyestradiol, a metabolite of estradiol-17b, decreased cell invasion and metastasis of gastric carcinoma through attenuation of Akt activity [66]. The bone morphogenetic protein signaling pathway enhances tumor invasiveness and metastasis in gastric carcinoma by sequential activation of PI3K/Akt followed by induction of NF-κB and MMP-9 activity [67]. Wnt5a dose-dependently stimulates the migration of human gastric carcinoma cells by enhancing phosphorylation of PI3K/Akt and GSK3β and by activating RhoA [68]. Notch2, a transmembrane receptor that mediates local cell-cell interaction in mammals, negatively regulates cell invasion and expression of MMP-9 by inhibiting the PI3K/Akt signaling pathway [69]. Micro-RNA (miRNA) miR-338-3p inhibits cell proliferation and clonogenicity and induces G1/S arrest and apoptosis in gastric carcinoma cells. Hyperactivation of miR-338-3p and silencing of P-Rex2a reduces the expression of P-Rex2a, resulting in the activation of PTEN and a decline in the phosphorylation of Akt [70]. miR-214 could also post-transcriptionally mediate PTEN expression by binding to the 3'-UTR of its mRNA. The expression level of miR-214 increases in gastric carcinoma and downregulates miR-214, leading to attenuated proliferation and tumor invasion [50].

## 5. The Role of the PI3K/Akt/mTOR Pathway in Resistance to Chemotherapy in Gastric Carcinoma

Anticancer agents and radiation therapy attack target cells by inducing apoptosis. In contrast, failure to activate apoptotic signaling represents an important mode of drug resistance in tumor cells [71]. Thus, resistance to chemotherapy is a major clinical problem in the treatment of all malignancies, including gastric carcinoma. The PI3K pathway is commonly activated in advanced gastric carcinoma [72,73] and survival signals induced by several receptors are mediated mainly by the PI3K/Akt/mTOR pathway [8]. A previous report has shown that LY294002 enhances the sensitivity of Fas-mediated apoptosis of gastric cancer cells by inducing p27/Kip accumulation and by dowregulating Mcl-1 expression [74]. LY294002 and vincristine were shown to synergistically promote growth inhibition, which was associated with decreased expression of MDR1/P-gp, Bcl-2, and XIAP, as well as with upregulation of Bax and caspase-3 expression [75]. BEZ235, a novel inhibitor of PI3K and mTOR, exerts antitumor effects against gastric carcinoma and enhances the effects of nab-paclitaxel through inhibition of cell proliferation and modulation of the PI3K/mTOR pathway [76]. Therefore, inhibition of PI3K/Akt activation may overcome the resistance of cancer cells to anticancer therapy. 

The use of etoposide and doxorubicin in gastric carcinoma enhanced Akt activity in a manner that was dependent on PI3K [77]. Akt activity was correlated with increased resistance to multiple chemotherapeutic agents such as 5-fluorouracil (5-FU), doxorubicin, and cisplatin [73]. NF-κB is another factor mediating chemoresistance induced by Akt. Chemotherapy facilitates the activation of NF-κB, thereby inhibiting the PI3K/Akt pathway [78]. PrP^C^ is a glycosylphosphatidylinositol-anchored membrane protein. Inhibition of PI3K/Akt signaling results in reduction of multidrug resistance of gastric carcinoma cells by downregulation of P-glycoprotein induced by PrP^C^ [79]. Upregulation of p-Akt confers resistance of gastric carcinoma cells to anticancer agents and suppresses adriamycin-induced apoptosis [80]. Inhibition of p-Akt expression reverses Akt-mediated multidrug resistance, upregulates p53 expression, and downregulates the expression of P-glycoprotein and transcription of multidrug resistance gene 1. The chemosensitivity of gastric carcinoma cells to cisplatin increased significantly after RNAi knockdown of Akt1 expression. This increase may be associated with the inactivation of the PI3K/Akt1 signaling pathway followed by the induction of Bax expression and a decrease in Bcl-2 expression [81].

Several studies have shown that PTEN is involved in drug resistance in various types of malignancies, including gastric carcinoma [82,83]. Downregulation of PTEN can lead to chemotherapeutic drug resistance, including DDP resistance, in gastric cancer patients [35,73]. Interestingly, the mTOR inhibitor everolimus specifically enhanced fluorouracil-induced apoptosis in two cell lines with HER2 amplification. This result suggests that mTOR inhibitors may be alternative drugs in gastric cancers with HER2 amplification [84].

Several other factors, including transcription factors, have been confirmed as mediators of chemoresistance through the PI3K/Akt/mTOR pathway in gastric carcinomas. Recent studies have suggested that miRNAs such as those in the miR-17 family, are novel candidate targets in the advancement of resistance to chemotherapy [85]. miR-106a, which belongs to the miR-17 family, is upregulated in DDP-resistant cells. Its overexpression regulates apoptosis and DDP resistance in gastric carcinoma cells. Enhanced expression of miR-106a also leads to downregulation of PTEN and more active signaling through the PI3K/Akt pathway. miR-21 also confers cisplatin resistance in gastric carcinoma cells by regulating PTEN [85]. Expression of miR-34a has been shown to be downregulated in cisplatin-resistant gastric carcinoma cell lines. miR-34a overexpression could improve the sensitivity of cells against cisplatin-based chemotherapies via the PI3K/Akt/survivin signaling pathway. miRNA-19a/b affects multidrug resistance in gastric carcinoma cells by targeting PTEN through regulation of Bcl-2 and Bax [86]. p21-activated kinase 4 (PAK4) induces CDDP resistance in gastric carcinoma cells by activation of the MEK/Erk and PI3K/Akt pathways. There exists mutual activation between the PAK4 and PI3K/Akt pathways in gastric carcinoma cells [87]. The forkhead transcription factor FOXO1 mediates cisplatin resistance in gastric carcinoma cells by activating the PI3K/Akt pathway. CDDP-induced FOXO1 activation is accompanied by an increase in p110α and p-Akt expression [88]. Interestingly, Tamoxifen reverses drug efflux transporters in P-gp-mediated multidrug resistance of human estrogen-receptor-negative gastric carcinoma cells by inhibiting the PI3K/Akt signaling pathway in these cells [89]. Activation of PI3K/Akt and NF-κB by TRAIL (TNF-related apoptosis-inducing ligand) confers resistance of human gastric cancer cells to TRAIL. Interference of survival signals significantly enhances apoptosis induced by TRAIL [90].

## 6. Targeting PI3K/Akt/mTOR as a Therapy for Gastric Carcinoma

**Figure 3:**
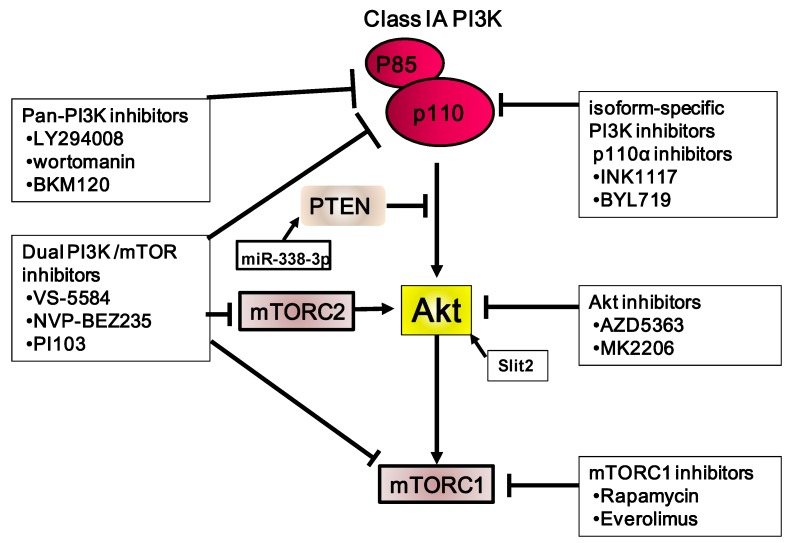
Summary of the PI3K/Akt/mTOR inhibitors used in gastric carcinoma, including those used in clinical trials. Stimulatory events are indicated by arrows and inhibitory events are indicated by lines ending in flat lines.

Inhibitors and antibodies targeting specific molecules, including PI3K pathways, are becoming more advanced; clinical trials have demonstrated some of these to be effective. The significant role of the PI3K/Akt/mTOR pathway in the initiation and development of gastric carcinoma suggests that this pathway may be an appropriate target for cancer therapy (Figure 3). Such therapy may involve inhibiting cell proliferation, enhancing apoptosis, and restoring the sensitivity of cancer cells to chemotherapy. Although these strategies are promising, clinical development of PI3K/Akt/mTOR pathway inhibitors still faces many problems [91]. PI3K inhibitors may be classified into several functional categories: PI3K inhibitors, dual mTOR1/mTOR2 inhibitors, Akt inhibitors, mTOR1 inhibitors, and dual PI3K/mTOR inhibitors. These inhibitors may exhibit differences in their affinity for different isoforms of these proteins. Derivatives of these inhibitors are more selective and are highly effective in targeting the PI3K/Akt/mTOR pathway, either alone or in combination.

### 6.1. PI3K Inhibitors

Several clinical trials targeting the PI3K/Akt/mTOR pathway are ongoing (Table 2). The first PI3K inhibitors identified were LY294002 [92] and wortmannin, a natural product from *Penicillium wortmannii* [93]. Our previous study showed that LY294002 reduces the total volume of metastatic nodules per mouse of OCUM-2MD3 in the peritoneal cavity in an experimental metastatic model [56]. However, these two inhibitors have been found to be toxic in animals. Several different types of PI3K inhibitors have also been tested against various tumor types in clinical trials. The combination of NVP-BKM120, a pan-class I PI3K inhibitor, and AG490, a STAT3 inhibitor, shows synergistic induction of apoptosis; however, this effect was observed only in cells harboring mutant KRAS. This result suggests that dual inhibition of signals may be an effective therapeutic strategy for KRAS-mutant gastric cancer patients [94]. An HER3-neutralizing antibody, LJM716, and an ATP-competitive p110α-specific inhibitor, BYL719, synergistically inhibit the growth of tumor xenografts. Gastric tumors in mice treated with a combination of these two inhibitors exhibited reduced p-Akt expression [95]. A phase I study on the safety of BYL719 and an HSP90 inhibitor, AUY922, in patients with advanced gastric cancer is ongoing.

**Table 2:** Clinical trials investigating target agents for gastric carcinoma.

Therapeutic Agent	Target	Clinical Trial	Efficacy	Year	Ref
Everolimus *vs.* placebo	mTORC1	Phase III (GRANITE-1)	PFS 1.68 *vs.* 1.41, *p* < 0.0001 OS 1.68 *vs.* 1.41, *p* = 0.1244	2013	[97]
Everolimus ± paclitaxel	mTORC1	Phase III (AIO-STO-0111)	Enrolling		
MK-2206 + Trastuzumab	Akt	Phase I	1 of 4 patient archive SD	2013	[97]
BYL719	p110α	Phase I	Enrolling		
BKM120	PI3K	Phase I	Enrolling		

PFS, progression free survival; OS, overall survival.

### 6.2. Akt Inhibitors

Several classes of Akt inhibitors are currently being developed. The effect of the Akt inhibitor, AZD5363, was determined according to the mutation of *PIK3CA* in gastric carcinoma cells. AZD5363 and taxotere administered in combination showed significant antitumor activity in PTEN loss in an *in vivo* model [98]. Targeting all Akt isozymes is better than inhibition of a single enzyme, although toxicity may be an issue. GSK690693 is an ATP-competitive, low-nanomolar, pan-Akt kinase inhibitor [99]. A potent, highly selective pan-Akt inhibitor, MK-2206, is now in early clinical trials [97]. Preliminary evidence shows that simultaneous administration of MK-2206 and trastuzumab has therapeutic efficacy in patients with HER2^+^ breast or gastroesophageal carcinoma, with a clinical benefit response rate of ~24% and a median time of progression of 72 days. The synergism of the two agents is as tolerable as the same dose of MK-2206 alone.

### 6.3. mTOR Inhibitors

Proof-of-principle that the PI3K pathway in cancer can be successfully targeted has been demonstrated by the development of rapamycin analogs that inhibit mTORC1 kinase (e.g., temsirolimus and everolimus) [100]. However, recent results have been disappointing when these analogs have been administered as single agents in gastric carcinoma.

Everolimus, a derivative of rapamycin, is an oral inhibitor of mTOR. Everolimus has been shown to be effective in several solid tumors, including gastric carcinomas. Several clinical trials have evaluated the efficacy and safety of this agent for gastric carcinoma. In a multicenter phase II study, everolimus monotherapy resulted in a promising DCR in patients with previously treated advanced gastric carcinoma. In general, this treatment is well-tolerated and has manageable side effects [101]. Based on these results, a randomized, double-blind phase III study (GRANITE-1) was performed to compare everolimus with BSC against placebo and against BSC in patients with gastric carcinoma [96]. Compared with BSC, everolimus did not significantly improve overall survival for patients with advanced gastric carcinoma that progressed after one or two sessions of previous systemic chemotherapy. Nevertheless, everolimus reduced the risk of progression by 34%, and the PFS was 1.7 *versus* 1.4 months, respectively, suggesting promise of combination therapy using everolimus with other molecular targeted or antitumor agents. Interestingly, MK-2206 and everolimus may be combined to synergistically inhibit PI3K/Akt/mTOR signaling and growth of gastric carcinoma cells. This enhancement is due to MAPK-dependent autophagy, and not to the apoptotic pathway [102].

### 6.4. Dual mTORC1/2 Inhibitors

Limiting mTORC1 inhibition correlates with the negative feedback loops that regulate the PI3K pathway controlled by mTORC1. Inhibition of mTORC1 alone can lead to a paradoxical increase in mTORC2 activity and thus continuation of Akt signaling [103]. A recent study revealed that rapamycin activates p-Akt and mTORC2 in gastric carcinoma cells. Downregulation of RICROR, which is regulated by mTORC2-induced Akt activation, reduces phosphorylation of GSK3 and cancer cell motility, suggesting that mTORC2 inhibition may abrogate the unfavorable signaling effects of mTOR inhibitors [104]. Therefore, mTORC1 and mTORC2 inhibition may be more effective in blocking the mTOR pathway Development of small-molecule inhibitors for mTORC1/2 has made progress. AZD2014, AZD8055, and OSI-027 are currently in clinical trials in patients with solid tumors. However, there is still no information about their activity in gastric carcinoma.

### 6.5. PI3K and mTOR Inhibitors

Inhibition of PI3K and mTOR1/2 results in inhibition of growth of gastric carcinoma cell lines greater than that attained with mono-inhibition alone. VS-5584 is a novel, low-molecular-weight compound with high and equivalent potency against mTOR and against PI3K class I isoforms. This compound has no activity against many lipid and protein kinases; therefore, it has a profile different from that of compounds in the clinical stages at present. Interestingly, VS-5584 was compared with 5-FU in a HER2-overexpressing gastric xenograft model. 5-FU and VS-5584 inhibited gastric carcinoma tumor growth by 32% and 121%, respectively, with a statistically significant difference for VS-5584 only [105]. Monotreatment of gastric-tumor-bearing mice with VS-5584 or with gefitinib resulted in tumor growth inhibition of 88% and 17%, respectively, with statistically significance for VS-5584 only. Combination therapy at the same dose levels resulted in tumor growth inhibition of 121%, with good tolerance for the therapy and no significant loss of body weight. The PI3K and mTOR inhibitor, PI103, acted in synergy with 5-FU in *PIK3CA*-mutated gastric carcinoma cells. This effect was associated with reductions in E2F1 and thymidylate synthase, and with increased DNA damage [106]. BEZ235 and BKM120 induces pro-apoptotic effects in all cell lines, with a particularly high response in *PI3KCA*-mutated gastric carcinoma cell lines [107]. On the other hand, cancers harboring KRAS mutations are likely to be insensitive to single-agent PI3K inhibitors and this type of malignancies show synergism in combination treatment with MEK inhibitors [108]. Thus, it is believed that combined inhibition is required to suppress the activation of other pathways and feedback-loop-induced activation of other signaling pathways and thereby to potentially lead to greater induction of apoptosis. Furthermore, BEZ235 has been shown to have antitumor effects against gastric carcinoma. It enhances the inhibitory effects of paclitaxel and reduces cell proliferation [76]. 

### 6.6. Other Therapeutic Approaches

Suppression of Akt1 and PIK3R1 expression using Akt1 and PIK3R1 small hairpin RNA RNAi can inhibit gastric carcinoma cell growth *in vitro* and *in vivo* [109]. Adenovirus PI3K(I) RNAi GFP induces mitochondrial dysfunction and activates apoptosis in gastric carcinoma cells, leading to increased formation of autophagosomes. Additionally, cells treated with adenovirus PI3K(I) RNAi GFP exhibit typical signs of autophagy [110].

Recent studies have shown that expression of HGF and its receptor c-Met is enhanced in gastric carcinoma. Thus, several therapeutic approaches targeting c-Met have been explored [111]. The small-molecule inhibitor, PHA-665752, which targets the catalytic activity of c-Met kinase, decreases PI3K signaling and correlates with potent cytoreductive activity in a gastric carcinoma xenograft model [112]. Moreover, knockdown of c-Met by RNA interference *in vitro* leads to reduction of Akt and to apoptotic cell death *in vitro* [113]. The c-Met inhibitor, KRC-408, exerts antitumor effects by directly affecting tumor cell growth and survival through attenuation of Akt and Erk phosphorylation as well CD34 expression in gastric tumors [114].

## 7. Conclusions and Future Perspectives

Deregulation of the PI3K/Akt/mTOR pathway is frequently encountered in gastric carcinoma. This crucial pathway plays important roles in tumor initiation and progression, including those in proliferative activity and in apoptosis. PI3K signaling is also commonly associated with the metastatic cascade in gastric carcinoma, which includes proteolytic activity, cytoskeletal remodeling, and resistance to chemotherapy. Although several aspects of tumor inhibition are not fully understood, numerous small molecule inhibitors targeting the PI3K/Akt/mTOR pathway are currently being studied in clinical trials for gastric carcinoma. Notably, gastric carcinoma is not a homogeneous disease and thus requires more personalized treatment. The heterogeneity of gastric carcinomas often causes delays in clinical trials for treatments against this cancer. One important approach to overcome this problem may be elucidation of feedback loops within the PI3k/Akt/mTOR pathway and of crosstalk with other signaling pathways [115]. Combination therapy with other cytotoxic or targeted agents and development of multitargeting agents may eliminate resistance and may result in the synergistic improvement of clinical benefits [116]. 

Trastuzumab has already been shown to have therapeutic utility in gastric carcinoma. The PI3K pathway is one of the main downstream signals of HER2. In gastric carcinoma, high expression levels of HER2 result in enhanced activity of Akt, which is closely related to poor prognosis. Consequently, PI3K pathway alterations are may be biomarkers for predicting the effectiveness of trastuzumab therapy. Understanding the specific association between HER2 expression and PI3K/Akt/mTOR pathway alterations in gastric carcinoma may lead to the development of new therapeutic strategies using trastuzumab. 

Considering that activation of PI3K/Akt/mTOR pathway is necessary for each event in the metastatic cascade, this pathway is likely to be a key underlying process for metastasis; therefore, focusing on this pathway may be worthwhile. Evaluation of the signaling networks and molecules that are involved in the metastasis of gastric carcinoma, for example, PI3K, may lead to novel therapeutic strategies. Management of metastasis is critical for good prognosis in patients. Rational use of biological therapies that have different molecular targets, such as integrin signaling and proteolytic factors, may have synergistic efficacy. 

Comprehensive knowledge of the PI3K/Akt/mTOR pathway would be useful in understanding the mechanism of tumor development and in identifying the ideal targeted anticancer therapy for gastric carcinoma. Appropriate clinical trials that incorporate predictive biomarkers need to be developed, as these may lead to personalized therapy. 
